# Adaptive Memory-Augmented Unfolding Network for Compressed Sensing

**DOI:** 10.3390/s24248085

**Published:** 2024-12-18

**Authors:** Mingkun Feng, Dongcan Ning, Shengying Yang

**Affiliations:** School of Information and Electronic Engineering, Zhejiang University of Science and Technology, Hangzhou 310023, China; 222308855056@zust.edu.cn (D.N.); syyang@zust.edu.cn (S.Y.)

**Keywords:** compressed sensing, proximal gradient descent, deep unrolling, neural networks, image reconstruction

## Abstract

Deep unfolding networks (DUNs) have attracted growing attention in compressed sensing (CS) due to their good interpretability and high performance. However, many DUNs often improve the reconstruction effect at the price of a large number of parameters and have the problem of feature information loss during iteration. This paper proposes a novel adaptive memory-augmented unfolding network for compressed sensing (AMAUN-CS). Concretely, without loss of interpretability, we integrate an adaptive content-aware strategy into the gradient descent step of the proximal gradient descent (PGD) algorithm, driving it to adaptively capture the adequate features. In addition, we extended AMAUN-CS based on the memory storage mechanism of the human brain and propose AMAUN-CS^+^ to develop the dependency of deep information across cascading stages. The experimental results show that the AMAUN-CS model surpasses other advanced methods on various public benchmark datasets while having lower complexity in training.

## 1. Introduction

Compressed sensing theory shows that when a signal is sparse or can be sparsely represented in a certain transform domain, the original signal can be recovered from the sampled signal at a much lower frequency than the Nyquist sampling frequency. CS is capable of sampling and compressing signals at the same time, which greatly alleviates the problem of wasted resources at the sampling end of the signal [1,2]. Since its proposal, this theory has been widely used in many fields, such as single-pixel imaging [3], cognitive radio networks [4], video compression [5], information security [6], speech and sound processing [7], radar imaging [8], low-dose CT [9], optical tomography [10], fluorescence microscopy [11], and MRI [12].

Mathematically, the purpose of CS reconstruction is to reconstruct the original signal $x\in {\mathbb{R}}^{N}$ from a random measurement $y\in {\mathbb{R}}^{M}$, which can be expressed as:

(1) y=Φx,

where $\Phi \in {\mathbb{R}}^{M\times N}$ represents the random measurement matrix, and r=M/N is defined as the sampling rate of CS. Since in CS M≪N, the reconstruction of X by y and Φ is an ill-posed inverse problem, this paper focuses on image reconstruction based on CS in natural scenes. Signal reconstruction is a difficult problem in CS theory, and traditional methods generally use some structured sparsity as an image prior, and then solve the sparsity regularization optimization problem in an iterative manner [13,14,15,16], which has the advantage of utilizing the a priori knowledge of the signal to model the signal, and thus possesses a certain degree of mathematical interpretability. However, its disadvantage is that the iterative algorithm leads to excessive computational complexity, while the need to manually select the optimal parameters limits the reconstruction performance of the CS algorithm.

In recent years, thanks to the powerful data processing capabilities of deep learning in computer vision [17], numerous CS methods have been proposed, which are broadly classified into two categories: (1) deep neural network-based methods; (2) optimization-inspired deep unfolding networks. Most existing deep neural network-based methods [18,19,20,21,22,23] achieve reconstruction by directly learning the mapping function between an image and its CS measurements, and this way of learning a priori from an external dataset ignores the internal imaging model, leading to its lack of flexibility in different imaging modalities. To address this problem, deep unfolding networks unfold traditional optimization algorithms into deep neural networks in order to improve the fidelity of the imaging model through a data-driven prior, achieving a balance between speed and interpretability.

However, most existing deep-learning reconstruction methods still have some shortcomings. For example, due to the weight-sharing nature of convolutional neural networks, the extracted features are mainly affected by the spatial distribution of pixels, thus making it difficult for the network to flexibly and adaptively adjust and learn according to the image content. In addition, many network frameworks are purely oriented to the reconstruction effect, and many stacked network layers are used to obtain better reconstruction performance, which also leads to a huge number of parameters and high computational costs. Finally, in order to enhance the information transfer capability between adjacent iterations, many deep unfolding networks tend to introduce residual feature information during the optimization process; however, in this way, the information transfer effect is limited, and the fault tolerance is poor. All these problems limit the further improvement of image reconstruction quality.

To cope with the above deficiencies, we propose an adaptive memory-augmented unfolding network for compressed sensing, in which we design a content-aware adaptive gradient descent module. The module adaptively adjusts the output result of algorithmic gradient descent by dynamic weights according to different scene elements, so as to obtain a feature gradient representation with stronger generalization. Meanwhile, we construct a lightweight dual-scale gated denoising module, which flexibly extracts useful information through the gating mechanism and denoises the image at different scales to improve the denoising performance. In addition, in order to be applicable to different application scenarios, this paper extends AMAUN-CS, based on which we utilize the memory storage mechanism of the human brain to design the deep memory transfer module (DMTM) for long-term memory of the information between neighboring iterations and name this network structure as AMAUN-CS^+^. Extensive experiments show that our proposed AMAUN-CS and AMAUN-CS^+^ have superior reconstruction performance compared with other CS methods on the Set11, Urban100, and BSD68 benchmark datasets.

Briefly, the main contributions of this paper can be summarized as follows:

- We propose a novel adaptive memory-augmented unfolding network for compressed sensing that can adaptively capture more features and recover more details and textures.
- We designed a content-aware adaptive gradient descent module (CAAGDM), which can flexibly extract information and adaptively adjust the output results. Meanwhile, we design a lightweight dual-scale gated denoising module (LDGDM), which effectively reduces the impact of different noises in network learning. In addition, we propose a DMTM, which aims to enhance the transfer and interaction of information in the optimization iteration phase.
- Extensive experimental results on several benchmark datasets show that the proposed AMAUN-CS and AMAUN-CS^+^ have superior reconstruction performance on the compressed sensing reconstruction task compared to most methods.

## 2. Related Work

### CS Reconstruction Approaches

Traditionally, the inverse problem of compressed imaging is usually solved by solving the following optimization method:

(2) $$x=\underset{x}{\mathrm{argmin}}\frac{1}{2}{\left\|\Phi x-y\right\|}_{2}^{2}+\lambda R(x),$$

where λ represents the regularization parameter, and R(x) denotes the regularization term associated with the prior knowledge of the image. This approach usually adopts an iterative optimization framework, e.g., approximate message passing (AMP) [13], iterative shrinkage thresholding algorithm (ISTA) [14], half-quadratic splitting (HQS) [15] algorithms, and the alternating direction multiplier method (ADMM) [16], which usually have good convergence and theoretical-analytical advantages but are often constrained by high computational complexity and low adaptivity.

In recent years, compressed sensing methods based on deep learning have been proposed one after another, demonstrating good reconstruction results and fast inference speeds. Shi et al. [18] proposed a method using convolutional layers of neural networks for the first time instead of the traditional hand-designed measurement matrices, which jointly optimizes the sampling and reconstruction networks. Fan et al. [19] proposed an improved multi-scale convolutional layer neural network model for input images for sampling. Regarding reconstruction, Kulkarni et al. [20] proposed an improved non-iterative reconstruction network (ReconNet) method based on convolutional neural networks, which achieves non-iterative reconstruction through weight sharing, thus reducing the computational complexity. Yao et al. [21] proposed a deep residual reconstruction network (DR^2^-Net) method, where DR2-Net uses a residual network to learn the inverse mapping between measurements and reconstructed images. However, all the above methods inevitably produce block artifacts in the reconstructed image. Thereafter, Zhou et al. [22] proposed a multi-channel block compression-based perceptual deep network (DeepBCS) method, which reduces the artifactual noise by using multi-channel block-by-block sampling and whole-image-based denoising. Tian et al. [23] proposed the use of generative adversarial networks combined with pixel loss to optimize the reconstruction results, which addresses the problem of over-smoothing and lack of structural information, such as texture after image reconstruction. This class of deep neural network methods lacks theoretical interpretability due to the black-box principle of its modeling, thus falling into a data-driven dependency, where the extent of its resource requirements is proportional to the data size.

Deep unfolding network maps the reconstruction iteration process of mathematical algorithms into a deep network model; Zhang et al. [24] proposed an optimization-inspired iterative shrinkage thresholding algorithm (ISTA). ISTA-Net combines the traditional iterative shrinkage thresholding algorithm with a convolutional neural network, and uses a nonlinear transform to solve the proximal mapping associated with the sparse-induced regularization factor to further improve the quality of reconstructed images. Thereafter, Zhang et al. [25] proposed an optimization-inspired interpretable deep network (OPINE-Net^+^) based on ISTA-Net. OPINE-Net+ performs deep reconstruction through the local similarity between image blocks, which effectively mitigates the block effect. Yang et al. [26] proposed a compression based on the alternating direction multiplier method for the compression sensing network (ADMM-CSNet), and ADMM-CSNet achieved remarkable results in MRI. Zhang et al. [27] developed a multilayer network from the denoising point of view by iteratively unfolding the approximate message-passing algorithm into a multilayer network. Recently, Shen et al. [28] proposed a deep unfolding network based on a hybrid of convolutional neural network (CNN) and Transformer, which combines the features of CNN and Transformer that can acquire both local and non-local information of the image and apply them to the steps of gradient descent and soft thresholding iterations of ISTA.

Although existing deep unfolding networks inherit good structure from optimization algorithms and have good interpretability, their prevalent design of single-channel images as inputs and outputs for each stage leads to the loss of many image details and greatly hinders information transfer in the network. Moreover, existing solutions lack flexibility in processing information and face high modeling complexity. In this paper, we propose an efficient solution.

## 3. Proposed Method

### 3.1. Overall Architecture

The proximal gradient descent (PGD) algorithm is well suited for solving many large-scale linear inverse problems [29], e.g., general CS and CS-MRI. Conventional PGD solves the compressed sensing reconstruction problem in Equation (2) by iteratively updating between the following two steps, and thus, the *k*th optimization process of the algorithm is denoted as:

(3) $$r^{\left(k\right)}=x^{\left(k-1\right)}-\rho^{\left(k\right)}\Phi^{⊤}(\Phi x^{\left(k-1\right)}-y),$$

(4) $$x^{\left(k\right)}=\underset{x}{\mathrm{argmin}}\frac{1}{2}{\left\|x-r^{\left(k\right)}\right\|}_{2}^{2}+\lambda R(x),$$

where *k* denotes the number of iterations, $x^{\left(k\right)}$ denotes the output image of the *k*th iteration, y denotes the sampled image, $\Phi^{T}$ denotes the transpose of the measurement matrix Φ, Equation (3) denotes the gradient descent step, $\rho^{\left(k\right)}$ is a learnable descent step size, and Equation (4) denotes the proximal mapping step.

**Figure 1:**
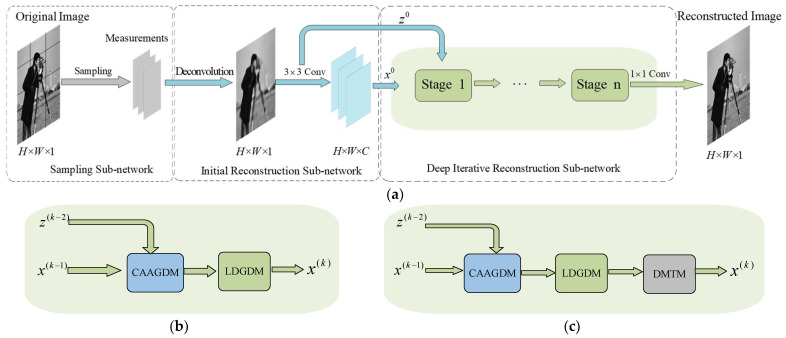
(**a**) Shows the overall AMAUN-CS framework; (**b**) AMAUN-CS *k*th optimization stage specific structure; (**c**) AMAUN-CS^+^ *k*th optimization stage specific structure.

As mentioned earlier, this traditional implementation lacks adaptivity and has information loss due to image-level inter-stage transmission. To rectify these weaknesses, we propose AMAUN-CS, which possesses a powerful data processing speed based on the deep neural network approach while fully retaining the core idea of the traditional iterative optimization algorithm to further enhance the theoretical interpretability of the network. The overall architecture of the network is shown in Figure 1a, and AMAUN-CS consists of a sampling subnetwork, an initial reconfiguration subnetwork, and a deep iterative reconfiguration subnetwork. Among them, the sampling sub-network replaces the traditional hand-designed measurement matrix with a convolutional layer, and the initial reconstruction sub-network converts the measurements in the low-dimensional space into the high-dimensional space to complete the initial image reconstruction. The reconstructed feature image is gradually optimized through multiple iterations in the deep iterative reconstruction subnetwork, and in each optimization stage, our designed CAAGDM adaptively adjusts the output result of the gradient descent step by using dynamic weights according to the different features of the inputs, and meanwhile, our proposed LDGDM is capable of denoising the image at two scales separately, thus improving the denoising performance. In order to effectively utilize the information of adjacent optimization stages, we further construct the DMTM, which enhances the information transfer capability between adjacent iterations through multiple gating mechanisms, and finally obtains the final reconstruction result using a 1 × 1 convolution after *n* optimization stages. Thus, as shown in Figure 1b, the *k*th iteration of AMAUN-CS can be expressed as ($k\in \left\{1,2,\ldots ,n\right\}$):

(5) $$r^{\left(k\right)}=H_{\mathrm{CAAGDM}}^{\left(k\right)}(x^{\left(k-1\right)},z^{\left(k-2\right)}),$$

(6) $$x^{\left(k\right)}=H_{\mathrm{LDGDM}}^{\left(k\right)}(r^{\left(k\right)}),$$

where $r^{\left(k\right)}$ and $x^{\left(k\right)}$ denote the *k*th feature domain output result, and Equation (5) represents the gradient descent process in the proximal gradient descent algorithm via our proposed CAAGDM. For the solution of Equation (4), it is well known that it actually corresponds to a denoising problem from a Bayesian perspective [30]. However, the hand-designed regularization term has some limitations because it relies on fixed threshold rules. Therefore, we implement Equation (6) by introducing the LDGDM, respectively, and $z^{\left(k-2\right)}\in {\mathbb{R}}^{H\times W\times C-1}$ is obtained by clipping latter C-1 channels from $x^{\left(k-2\right)}$. For the first iteration, the input $x^{\left(0\right)}$ is the feature result obtained by the 3 × 3 convolution of the initial reconstruction result.

### 3.2. Sampling and Initial Reconfiguration of Subnetworks

Instead of the traditional hand-designed measurement matrix for non-overlapping chunk sampling [18], we utilize a convolutional layer to first divide the input original image *x* into non-overlapping image chunks of size B×B, and then construct $r\times B^{2}$ unbiased term convolutional kernels $\Phi_{B}$ of size B×B and step size B×B. Thus, the sampling network can be represented as:

(7) $$y=\Phi_{B}*x,$$

where ∗ denotes the convolution operation and y denotes the measured value.

In order to make full use of the a priori information of the image, in the initial reconstruction stage, we use the transpose matrix ${\Phi_{B}}^{T}$ of $\Phi_{B}$ to back-convolve y to obtain the initial reconstructed image $x_{\mathrm{init}}$; ${\Phi_{B}}^{T}$ is actually the $B^{2}$ unbiased term convolution kernels of size $1\times 1\times rB^{2}$. Through the end-to-end training approach, the measurement matrix $\Phi_{B}$ and the reconstruction network parameters are able to be continuously learned in a data-driven manner, and therefore, the initial reconstruction can be expressed as:

(8) $$x_{\mathrm{init}}={\Phi_{B}}^{T}*y,$$

### 3.3. Deep Iterative Reconfiguration Subnetwork

#### 3.3.1. Content-Aware Adaptive Gradient Descent Module

**Figure 2:**
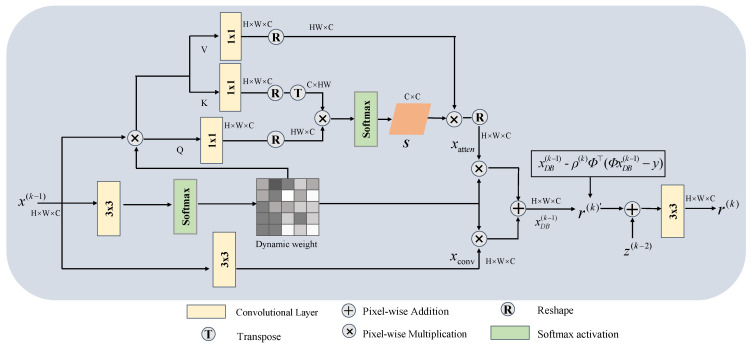
Illustration of the content-aware adaptive gradient descent module.

Before performing the gradient descent step, considering the existence of different scene elements in natural images, the variability of the optimal gradient backpropagation for each pixel, and the lack of flexible, adaptive adjustment of the existing network structure with respect to the image content [31], as shown in Figure 2, our proposed CAAGDM contains an attention branch and a convolutional branch. In the attention branch, the module utilizes the non-local self-similarity of the input features to generate model parameters with adaptive weights, which enables the effective extraction of key features in the image. The output results of the two branches are adaptively balanced by dynamic weights to enhance the model’s perceptual ability.

We first use a 3 × 3 convolution and activation function on the input image to obtain its dynamic weights, so the process can be represented as follows:

(9) $$\mathrm{dw}=\delta (W_{p}^{3}(x^{\left(k-1\right)})),$$

where δ denotes the Softmax activation function, $W_{p}^{3}$ denotes the convolution kernel size of 3 × 3, and dw denotes the dynamic weights.

Next, the shallow features are extracted using 3 × 3 convolution in the convolution branch, and the result is denoted as $x_{\mathrm{conv}}\in {\mathbb{R}}^{H\times W\times C}$, then the product of $x^{\left(k-1\right)}$ and the dynamic weight dw are used as the input to the attention branch, assuming that the input data are shaped as $x^{\left(k-1\right)}\in {\mathbb{R}}^{H\times W\times C}$, denoting that the channel of the input data are C, the height is H, and the width is W. The query (Q), key (K), and value (V) matrices are obtained by using the convolution on them, respectively, and then matrix dimensioning is performed on them to obtain $Q\in {\mathbb{R}}^{\mathrm{HW}\times C}$ and $K\in {\mathbb{R}}^{C\times \mathrm{HW}}$, respectively. After that, K is multiplied by Q and then by the activation function to obtain the attention matrix $s\in {\mathbb{R}}^{C\times C}$. Multiplying the attention matrix with V to capture the non-local self-similarity result of each feature, this output feature is denoted as $x_{\mathrm{atten}}\in {\mathbb{R}}^{H\times W\times C}$, and the process can be represented as:

(10) $$S=\delta (R(W_{p}^{1}(k^{T}))⊗R(W_{p}^{1}\left(Q))\right),$$

(11) $$x_{\mathrm{atten}}=R(W_{p}^{1}\left(k)\right)⊗S,$$

where R denotes Reshape, $W_{p}^{1}$ denotes convolutional kernel size of 1 × 1, and ⊗ denotes elemental multiplication.

Finally, the output result $x_{\mathrm{DB}}^{\left(k-1\right)}$ is adaptively balanced by dynamic weights for the two-branch output result, a process that is able to discard redundant features and focus on retaining important features. These optimized features are integrated into the gradient descent step in Equation (13) to obtain the output result $r^{(k)\prime }$. In addition, in order to enhance the ability of information interaction between adjacent iterations, CAAGDM fuses $z^{(k-2)}$ in the output stage to obtain the final result $r^{\left(k\right)}$. Therefore, the process can be expressed as:

(12) $$x_{\mathrm{DB}}^{\left(k-1\right)}=x_{\mathrm{conv}}⊗\mathrm{dw}+\mathrm{dw}⊗x_{\mathrm{atten}},$$

(13) $$r^{(k)\prime }=x_{\mathrm{DB}}^{\left(k-1\right)}-\rho^{\left(k\right)}\Phi^{⊤}(\Phi x_{\mathrm{DB}}^{\left(k-1\right)}-y),$$

(14) $$r^{\left(k\right)}=W_{p}^{3}(z^{(k-2)}+r^{(k)\prime }),$$

#### 3.3.2. Lightweight Dual-Scale Gated Denoising Module

The process of solving the proximal mapping can be regarded as denoising the image signal. However, traditional denoising methods tend to remove high-frequency texture information from the input image [32]. CNN-based methods have been shown to be very effective in denoising, but due to the weight-sharing inductive bias of convolutional neural networks, they tend to treat all input features as valid pixel features, which limits their flexibility. Considering the denoising effect and computational complexity, we designed a lightweight dual-scale gated denoising module that processes noise reduction on two scales, separately, to enhance overall performance. Meanwhile, a gating mechanism [33] is introduced in each scale to effectively suppress less informative features and allow only useful information to be passed further in the network optimization structure.

**Figure 3:**
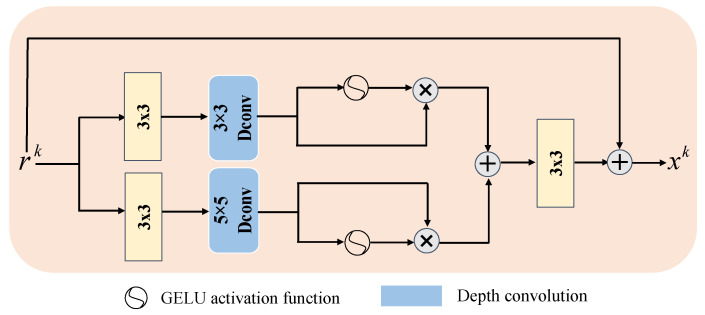
Illustration of the lightweight dual-scale gated denoising module.

LDGDM is shown in Figure 3. First, the input data are dimensionally expanded using 1 × 1 convolution, after which depth convolution is introduced to effectively capture the information of spatially neighboring pixels. Next, the features are divided into two branches, one of which uses the GELU activation function to realize the gating mechanism. Then, the features are multiplied element by element with the features of the other branch. Finally, the output features at each scale are fused using 1 × 1 convolution, thus filtering low-information features at different scales and retaining only useful information. In addition, the head section of the LDGDM uses hopping connections to map features to the residual domain, which facilitates information flow while further improving the denoising performance of the module, and thus can be expressed as follows:

(15) $$x^{k}=W_{p}^{3}(\mathrm{Gating}\;(r^{k}))+r^{k},$$

(16) $$\mathrm{Gating}(r^{k})=\sigma (W_{d}^{3}(W_{p}^{3}(r^{k})))⊗W_{d}^{3}(W_{p}^{3}(r^{k}))+\sigma (W_{d}^{5}(W_{p}^{3}(r^{k})))⊗W_{d}^{5}(W_{p}^{3}(r^{k})),$$

where σ denotes the GELU activation function, i.e., the gating mechanism, and $W_{d}^{3}$ and $W_{d}^{5}$ denote the depth convolution of 3 × 3 and 5 × 5 size, respectively.

### 3.4. Enhanced Version: AMAUN-CS^+^

**Figure 4:**
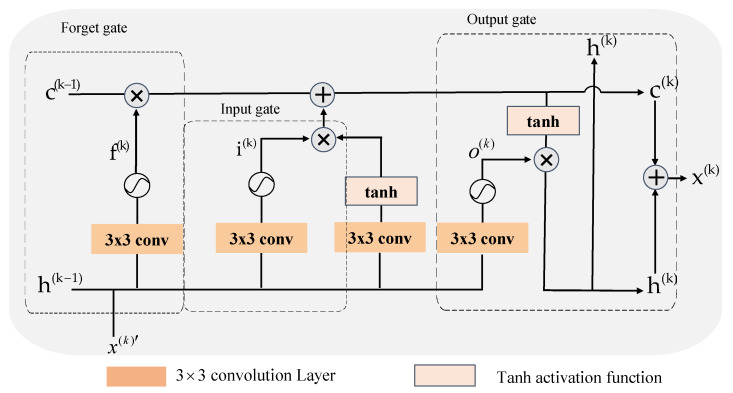
Illustration of the deep memory transfer module.

The human brain can realize the memory mechanism by continuously encoding and storing new information, which contributes to the formation and long-term storage of memories by enhancing the synaptic strength and connections between neurons in the brain [34]. Therefore, an optimization stage of a deep unfolding network can be regarded as a neuron in the human brain, and the information flow between adjacent iterations can be regarded as a memory transfer mechanism. Conventional optimization processes usually introduce residual feature information to enhance the information transfer capability between adjacent iterations. However, this approach is regarded as a kind of short-term memory, which is prone to losing information during multiple iterations. Thanks to the fact that a long short-term memory network (LSTM) can effectively capture long-term dependencies in sequential data [35], we designed a deep memory transfer module based on AMAUN-CS for long-term memorization of information across iterations, and the specific structure is shown in Figure 4. Therefore, the AMAUN-CS+ of the kth iteration can be expressed as:

(17) $$r^{\left(k\right)}=H_{\mathrm{CAAGDM}}^{\left(k\right)}(x^{\left(k-1\right)},z^{\left(k-2\right)}),$$

(18) $$x^{(k)\prime }=H_{\mathrm{LDGDM}}^{\left(k\right)}(r^{\left(k\right)}),$$

(19) $$x^{\left(k\right)}=H_{\mathrm{DMTM}}^{\left(k\right)}(x^{(k)\prime }),$$

where Equations (18) and (19) contain modules consistent with those described in Section 3.2 and Section 3.3, and this section is followed by a description of the manner in which the DMTM is implemented.

The core of DMTM is composed of a memory unit $c^{\left(k\right)}$ and three gating mechanisms, which are the forgetting gate, input gate, and output gate, and the input features can be memorized by the memory unit in the network for a long time, as shown in Figure 4, where the forgetting gate decides whether to keep the memory of the previous moment to the current moment, the input gate decides the effect of the input of the current moment on the memory, and the output gate decides the memory’s effect on the output $h^{\left(k\right)}$, which is the hidden state of the DMTM for storing previous memory information with an initial value of zero; $x^{(k)\prime }$ denotes the input image.

First, the DMTM extracts the information with convolution for the input image $x^{(k)\prime }$ and the hidden state $h^{\left(k\right)}$, respectively. It then obtains the information of the input gate through the sigmoid activation function. $f^{\left(k\right)}$ denotes the output of the forgetting gate, which discards the useless information by controlling the information retained by the previous memory unit $c^{\left(k-1\right)}$. The above process can be represented as follows:

(20) $$i^{\left(k\right)}=\sigma (W_{p}^{3}(x^{(k)\prime })+W_{p}^{3}(h^{\left(k-1\right)})+b_{i}),$$

(21) $$f^{\left(k\right)}=\sigma (W_{p}^{3}(x^{(k)\prime })+W_{p}^{3}(h^{\left(k-1\right)})+b_{f}),$$

where σ denotes the activation function and $b_{i}$, $b_{f}$ denote the bias terms of the input and forgetting gates, respectively.

Secondly, update the memory unit $c^{\left(k\right)}$; this process is mainly divided into two steps. The first step is to discard the useless information in the previous stage through the output $f^{\left(k\right)}$ of the forgetting gate, and the second step is to multiply the information $i^{\left(k\right)}$ of the input gate and the result obtained from the tanh function to selectively add useful information, this process can be expressed as follows:

(22) $$c^{\left(k\right)}=i^{\left(k\right)}⊗\tan h(W_{p}^{3}(x^{(k)\prime })+W_{p}^{3}(h^{\left(k-1\right)})+b_{c})+f^{\left(k\right)}⊗c^{\left(k-1\right)},$$

where $b_{c}$ denotes the bias term of the memory cell.

Finally, the value of the output gate $o^{\left(k\right)}$ and the hidden state $h^{\left(k\right)}$ are calculated, and the memory cell $c^{\left(k\right)}$ is further extracted with information using the tanh function. Then, the extracted result is multiplied by $o^{\left(k\right)}$ to obtain the result of $h^{\left(k\right)}$, and finally, the information of $h^{\left(k\right)}$ and $c^{\left(k\right)}$ is combined to obtain as $x^{\left(k\right)}$. This process can be expressed as follows:

(23) $$o^{\left(k\right)}=\sigma (W_{p}^{3}(x^{(k)\prime })+W_{p}^{3}(h^{\left(k-1\right)})+b_{o}),$$

(24) $$h^{\left(k\right)}=o^{\left(k\right)}⊗\tan h(c^{\left(k\right)}),$$

(25) $$x^{\left(k\right)}=h^{\left(k\right)}+c^{\left(k\right)},$$

where $b_{o}$ denotes the bias term of the output gate.

### 3.5. Loss Function

Given a set of fully sampled images ${\left\{x_{i}\right\}}_{i=1}^{N_{a}}$ and some sampling patterns with the specific CS ratio r, the training data ${\left\{y_{i},x_{i}\right\}}_{i=1}^{N_{a}}$ are generated by $y_{i}=\Phi x_{i}$, which will denote the input features $x_{i}$ and  x^i as well as denote the output reconstruction results. We adopt the *L*_1_ loss function to compute the difference between $x_{i}$ and  x^i, as shown below:

(26) L(Θ)=1NbNa∑i=1Naxi- x^i1,

where N_a_ and N_b_ denote the number of training images and the size of each image, respectively, and Θ denotes the set of all learnable network parameters, $\Theta ={\left\{\Phi_{B},H_{\mathrm{CAAGDM}\;}^{\left(k\right)}(\cdot ),H_{\mathrm{LDGDM}}^{\left(k\right)}(\cdot ),H_{\mathrm{DMTM}}^{\left(k\right)}(\cdot )\right\}}_{K=1}^{n}$.

## 4. Experimental Results and Analysis

### 4.1. Implementaion Details

We use the BSD400 [36] and DIV2K [37] datasets as training datasets. To achieve data enhancement, we obtained better generalization by randomly cropping, panning, and rotating the training set, setting the default number of training batches to 1500 and the batch size to 8. The network was trained using the Adam [38] optimizer with the initial learning rate set to 5 × 10^−4^ and the gradient descent step size $\rho^{\left(k\right)}$ as a learnable linear scaling factor, which is initialized to 0.5 in this paper. Our test set uses three public benchmark datasets: Set11 [20], BSD68 [39], and Urban100 [40]. Color images are processed in the YCbCr space and evaluated on the luminance channel. The reconstructed image quality evaluation criteria use objective metrics: peak signal-to-noise ratio (PSNR) [41], structural similarity (SSIM) [42], and subjective visual judgments of the human eye, and the experimental environment is an Intel Core i7-13700KF CPU and an RTX4070Ti GPU.

### 4.2. Evaluation Metrics

PSNR is a classical objective evaluation metric, the higher the PSNR value, the smaller the difference between the reconstructed image and the original image, i.e., the higher the image quality. For images X and Y, both of size m×n, the PSNR is computed as shown in Equation (27):

(27) $$\mathrm{PSNR}=10\cdot \log_{10}(\frac{{\mathrm{MAX}}_{X}^{2}}{\mathrm{MSE}}),$$

where ${\mathrm{MAX}}_{X}^{2}$ is the maximum possible pixel value of image X, and MSE denotes the mean square error between images X and Y.

SSIM is an objective evaluation metric used to measure the similarity between two images. The SSIM value ranges from 0 to 1, where the larger value indicates a higher similarity between the images. The SSIM between images X and Y is calculated according to Equation (28):

(28) $$\mathrm{SSIM}(X,Y)=\frac{\left(2\mu_{X}\mu_{Y}+C_{1})(2\sigma_{XY}+C_{2}\right)}{\left({\mu_{X}}^{2}+{\mu_{Y}}^{2}+C_{1})({\sigma_{X}}^{2}+{\sigma_{Y}}^{2}+C_{2}\right)},$$

where $\mu_{X}$ and $\mu_{Y}$ represent the mean values of images X and Y, while ${\sigma_{X}}^{2}$ and ${\sigma_{Y}}^{2}$ represent their variances. $\sigma_{XY}$ denotes their covariance. Additionally, $C_{1}$ and $C_{2}$ are constant terms.

### 4.3. Comparison with State-of-the-Arts

To validate the performance of AMAUN-CS, we compare it with recent representative CS reconstruction methods including TVAL3 [16], ReconNet [20], DPA-Net [43], ISTA-Net^+^ [24], AMP-Net [27], OPINE-Net^+^ [25], COAST [44], TransCS [28], SODAS-Net [45], and DPC-DUN [46]. Among them, TVAL3 is based on traditional optimization methods, ReconNet and DPA-Net are deep neural network methods, and all other methods are optimization-inspired deep unfolding networks. Table 1 summarizes the PSNR/SSIM reconstruction metrics of different methods on the Set11 dataset, and the boldfaced font shows the optimal value of each row. From Table 1, it can be observed that the objective metrics of AMAUN-CS and AMAUN-CS^+^ proposed in this paper are overall better than the other methods on all datasets.

As shown in Table 1, the average PSNR of AMAUN-CS and AMAUN-CS^+^ is improved by 0.9 dB and 1.07 dB, respectively, and the average SSIM is improved by 0.0087 and 0.0113 compared with the best performance of the other methods, TransCS. In addition, in order to further validate the generalization of the method of this paper, this paper compares the other methods with AMAUN-CS on the Urban100 and BSD68 for comparison, as shown in Table 2 and Table 3, the proposed method in this paper still significantly outperforms the other methods in PSNR/SSIM. In the Urban100 dataset, AMAUN-CS achieves an average improvement of 0.47 dB/0.0146 over the best-performing method, DPC-DUN, while in the BSD68 dataset, AMAUN-CS demonstrates an average improvement of 0.5 dB/0.0215 over the top-performing TransCS. By incorporating long- and short-term memory operations, AMAUN-CS^+^ further enhances performance based on AMAUN-CS, with an additional improvement of 0.35 dB/0.0036 and 0.24 dB/0.0049, respectively.

**Figure 5:**
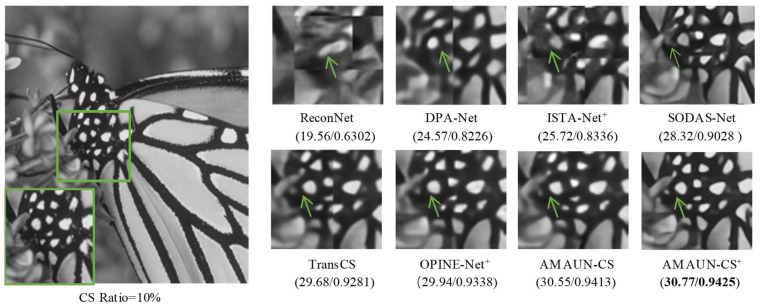
Comparisons on recovering an image from Set11 dataset in the case of CS ratio = 10%. The original image is marked with a green box, and the reconstructed image is marked with green arrows.

**Figure 6:**
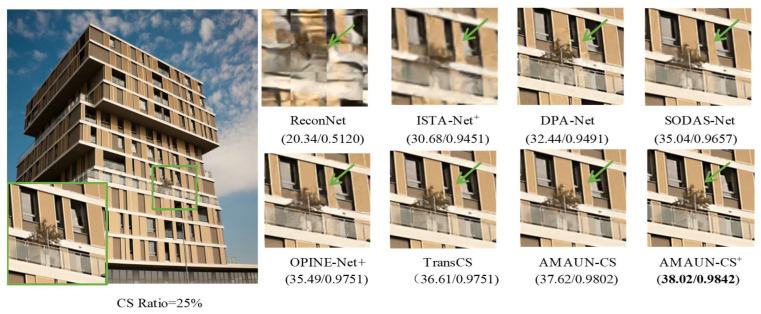
Comparisons on recovering an image from Urban100 dataset in the case of CS ratio = 25%. The original image is marked with a green box, and the reconstructed image is marked with green arrows.

In order to compare the differences in reconstruction results between AMAUN-CS and other methods based on subjective visualization, we present the following analysis. Figure 5 and Figure 6 show the reconstruction results of different methods at different observation rates. As shown in Figure 5, both ReconNet and ISTA-Net^+^ fail to reconstruct the image with complete details, resulting in a significant block effect. At the same time, TransCS and OPINE-Net^+^ make the reconstructed image blurrier and lose part of the texture information due to the lack of further utilization of the information between adjacent iterations.

**Figure 7:**
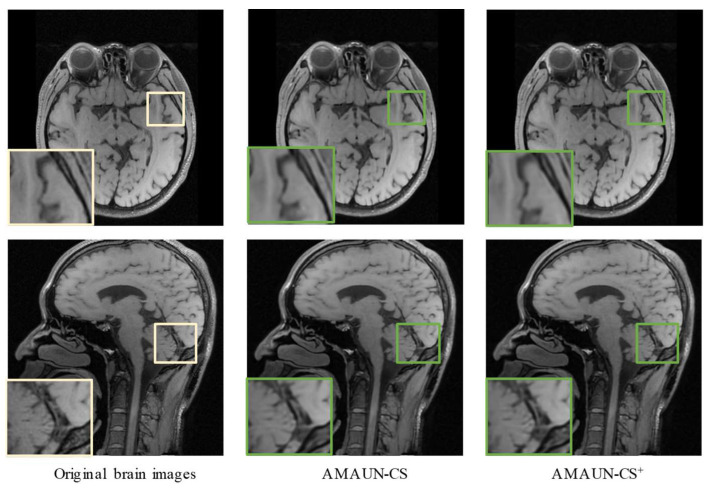
Comparison of image recovery from original brain images at a CS ratio of 25%. The original image is marked in gold, and the reconstructed image is marked in green.

In addition, Figure 6 gives a comparison of the reconstructed images of different methods on the color image Urban100. It can be seen that the other methods suffer from different degrees of block artifacts and blurring. Whereas the method proposed in this paper is more full in terms of texture details. It also recovers the structural information of the image better and makes the texture details more visible. In summary, the experimental results above demonstrate that the AMAUN-CS and AMAUN-CS+ methods proposed in this paper achieve advanced performance in both objective quality evaluation and subjective visual comparison. To assess the scalability of AMAUN-CS, a set of medical brain images was used to evaluate its performance on the CS-MRI reconstruction task. As shown in Figure 7, the experimental results demonstrate that our method can clearly reconstruct texture structures.

### 4.4. Complexity Analysis

The computation cost and model size are important in many practical applications. Table 4 compares the number of parameters and average running time of different methods in the 0.1 sampling rate and Set11 dataset. For the fairness of the comparison, only the algorithm complexity is compared with the open-source model on the same platform. As shown in Table 4, TransCS has a larger number of parameters and a relatively complex structure due to the dual-path network structure that combines CNN and Transformer. In contrast, the number of parameters of the network in this paper is less than that of other methods, and at the same time, it can maintain better reconstruction performance. It shows that the parameters of the model can be more fully utilized under the framework of this paper.

### 4.5. Ablation Analysis

#### 4.5.1. Number of Optimization Phases of the Network

**Figure 8:**
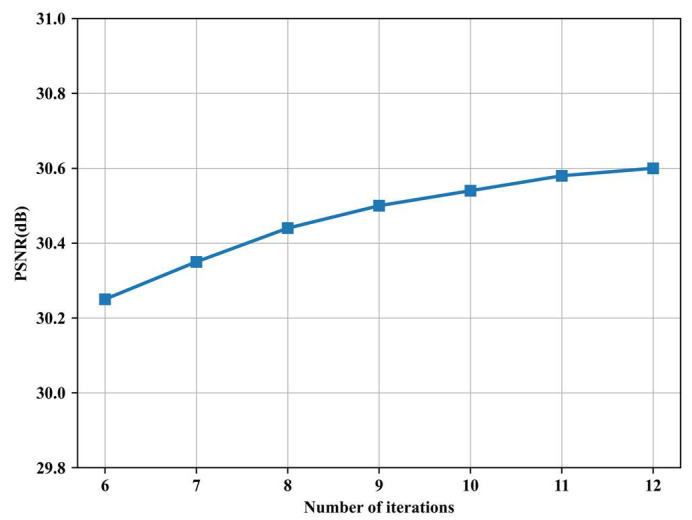
Performance of AMAUN-CS over various number of iterations.

To explore the effect of different iteration numbers on the image reconstruction results, Figure 8 shows the reconstruction performance of AMAUN-CS obtained by training with different numbers of iterations under the Set11 dataset and a 10% sampling rate. The horizontal axis is the number of training iterations of the network, and the vertical axis is the average PSNR value of the dataset reconstruction. From the curve, we observe that the PSNR value increases with the number of iterations, but it tends to plateau when n ≥ 9. While larger iteration numbers might further improve performance slightly, they also lead to increased computational complexity and parameter overhead. Therefore, we selected nine as the default iteration number, considering the trade-off between reconstruction performance and network complexity.

#### 4.5.2. Effectiveness of Different Modules

In order to validate the effectiveness of our proposed different network modules in AMAUN-CS, this section performs ablation experiments on three modules at a 10% sampling rate. As shown in Table 5, case (a) serves as our baseline model, with a simple CNN replacing our proposed module. In cases (b) and (c), CAAGDM and LDGDM are introduced alone, respectively, and gains of 2.06 dB/0.0075 and 1.48 dB/0.0036 are obtained compared to the baseline model, indicating the limited effect of LDGDM alone. Case (d) shows that when both sub-modules are used simultaneously, a significant improvement of 2.94 dB/0.0124 is achieved compared to the baseline model. Case (e) shows that the introduction of DMTM brings a further improvement of 3.09 dB/0.0153, which is good proof of its transfer of information across iterations.

In summary, the application of CAAGDM, LDGDM, and DMTM has a significant positive effect on improving the reconstruction of the baseline model.

### 4.6. Sensitivity to Noise

**Figure 9:**
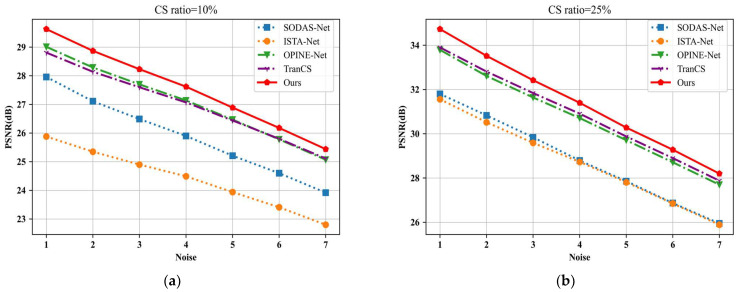
(**a**) The Gaussian noise robustness of the different methods is compared with a CS ratio of 10%; (**b**) the Gaussian noise robustness of the different methods is compared with a CS ratio of 25%.

To evaluate the robustness of the reconstructed images under different noise levels, we first added different levels of Gaussian noise to the original images of the Set11 dataset. AMAUN-CS and other methods were then sampled and reconstructed with the noisy images as inputs at 10% and 25% observation rates. Figure 9 illustrates the PSNR relationship of the various methods for different noise standard deviations. It can be seen that the image reconstruction quality of ISTA-Net is significantly affected and only slightly improved even at higher observation rates. The other methods are also affected by different degrees of noise interference at low observation rates. In contrast, the proposed method shows strong robustness under different noise levels.

## 5. Conclusions

In this paper, an adaptive memory-augmented unfolding network for compressed sensing was proposed. Specifically, the model effectively combines a proximal gradient descent algorithm with a deep neural network and designs a deep reconstruction sub-network with multiple phases to further refine the initial reconstructed image, with each optimization stage corresponding to one iteration of the traditional optimization algorithm. Meanwhile, oriented to lightweight, we then construct two learning modules, CAAGDM and LDGDM, to enhance the flexibility of the network, which effectively reduces the influence of different noises in network learning. In addition, we propose a deep memory transfer module to enhance the transfer and interaction of information in the optimization iteration phase. In extensive experiments on several benchmark datasets, we demonstrate its significant advantages over other state-of-the-art networks. In the future, we plan to extend AMAUN-CS to video transmission applications.

## Figures and Tables

**Table 1:** Average PSNR (dB)/SSIM performance comparisons of recent deep network-based CS methods on Set11 dataset with different CS ratios. The best and second-best results are highlighted in bold and underlined, respectively.

Methods			CS Ratio				
	4%	10%	25%	30%	40%	50%	Average
TVAL3	18.75/0.3997	22.99/0.4758	27.92/0.7238	29.00/0.7764	31.46/0.8531	33.55/0.8957	27.28/0.6874
ReconNet	20.93/0.5897	23.96/0.7172	26.38/0.7883	28.20/0.8424	30.02/0.8837	30.62/0.8983	26.68/0.7857
ISTA-Net^+^	21.32/0.6037	26.58/0.8066	32.48/0.9242	33.81/0.9393	36.04/0.9581	38.06/0.9706	31.37/0.8670
DPA-Net	23.50/0.7205	27.66/0.8530	32.38/0.9311	33.35/0.9425	35.21/0.9580	36.80/0.9685	31.48/0.8955
AMP-Net	25.26/0.7722	29.40/0.8779	34.63/0.9481	36.03/0.9586	38.28/0.9715	40.34/0.9804	33.99/0.9181
OPINE-Net^+^	25.69/0.7920	29.79/0.8905	34.81/0.9503	36.04/0.9600	37.96/0.9633	40.17/0.9797	34.07/0.9228
COAST	**-/-**	28.69/0.8618	32.54/0.9251	35.04/0.9501	37.13/0.9648	38.94/0.9744	**-/-**
TransCS	25.41/0.7883	29.54/0.8877	35.06/0.9548	35.62/0.9588	38.46/0.9737	40.49/0.9815	34.09/0.9241
SODAS-Net	24.69/0.7838	28.84/0.8665	34.24/0.9443	35.54/0.9545	37.72/0.9680	39.59/0.9769	33.43/0.9156
DPC-DUN	25.20/0.7710	29.40/0.8798	34.69/0.9482	35.88/0.9570	37.98/0.9694	39.84/0.9778	33.83/0.9172
AMAUN-CS	26.34/0.8089	30.50/0.9018	35.84/0.9596	37.08/0.96 68	39.19/0.976 7	41.00/0.9830	34.99/0.9328
AMAUN-CS^+^	**26.52/0.8122**	**30.72/0.9045**	**36.02/0.9614**	**37.26/0.9696**	**39.30/0.9792**	**41.15/0.9859**	**35.16/0.9354**

**Table 2:** Average PSNR (dB)/SSIM performance comparisons of recent deep network-based CS methods on Urban100 dataset with different CS ratios. The best and second-best results are highlighted in bold and underlined, respectively.

Methods			CS ratio				
	4%	10%	25%	30%	40%	50%	Average
TVAL3	18.15/0.4291	22.31/0.4655	27.75/0.7628	28.84/0.8214	31.74/0.8846	34.64/0.9315	27.24/0.7158
ReconNet	20.37/0.5514	23.74/0.7044	26.25/0.8402	28.07/0.8929	30.62/0.9131	32.98/0.9394	27.01/0.8069
ISTA-Net^+^	19.66/0.5370	19.66/0.7238	28.93/0.8840	30.21/0.9079	32.43/0.9377	34.43/0.9571	27.55/0.8245
DPA-Net	21.64/0.6498	24.55/0.7841	28.80/0.8944	29.47/0.9034	31.09/0.9311	32.08/0.9447	27.93/0.8513
AMP-Net	21.89/0.6340	26.04/0.8151	30.89/0.9202	32.19/0.9365	34.37/0.9578	36.33/0.9712	30.28/0.8724
OPINE-Net^+^	23.00/0.7020	26.93/0.8397	31.86/0.9308	32.58/0.9414	33.88/0.9445	37.23/0.9741	30.91/0.8882
COAST	**-/-**	25.94/0.8035	31.10/0.9168	32.23/0.9321	34.22/0.9530	35.99/0.9665	**-/-**
TransCS	23.23/0.7018	26.72/0.8413	31.72/0.9330	31.95/0.9381	35.22/0.9648	37.20/0.9734	31.00/0.8920
SODAS-Net	22.69/0.6991	25.65/0.7739	31.49/0.9190	32.90/0.9376	35.09/0.9583	37.23/0.9736	30.84/0.8769
DPC-DUN	23.02/0.7023	26.99/0.8345	32.36/0.9323	33.53/0.9449	35.61/0.9624	37.52/0.9737	31.51/0.8916
AMAUN-CS	23.89/0.7354	27.98/0.8684	32.54/0.9450	33.30/0.9435	36.76/0.9729	37.42/0.9724	31.98/0.9062
AMAUN-CS^+^	**24.04/0.7411**	**28.07/0.8710**	**3** **2.** **75/0.94** **68**	**3** **3.** **61/0.9** **457**	**37.01/0.9755**	**38.52/0.9784**	**32.33/0.9098**

**Table 3:** Average PSNR (dB)/SSIM performance comparisons of recent deep network-based CS methods on BSD68 dataset with different CS ratios. The best and second-best results are highlighted in bold and underlined, respectively.

Methods			CS Ratio				
	4%	10%	25%	30%	40%	50%	Average
TVAL3	17.86/0.4261	19.26/0.4758	21.27/0.7204	22.34/0.7764	25.39/0.8531	29.59/0.8957	22.62/0.6912
ReconNet	21.66/0.4994	23.88/0.6400	25.75/0.7317	26.72/0.7870	28.96/0.8499	30.13/0.8499	25.87/0.8499
ISTA-Net^+^	22.17/0.5486	25.32/0.7022	29.36/0.8525	30.20/0.8771	32.21/0.9321	34.04/0.9424	28.88/0.8095
DPA-Net	23.27/0.6096	25.02/0.6958	28.73/0.8814	29.93/0.8722	31.85/0.9128	33.60/0.9401	28.73/0.8186
AMP-Net	25.40/0.6985	27.86/0.7926	31.74/0.9048	32.84/0.9240	33.10/0.9383	35.02/0.9510	30.99/0.8682
OPINE-Net^+^	25.00/0.6825	27.82/0.8045	31.51/0.9061	32.35/0.9215	33.45/0.9412	36.47/0.9669	31.10/0.8704
COAST	-/-	26.28/0.7422	29.00/0.8413	31.06/0.8934	32.93/0.9267	34.74/0.9497	-/-
TransCS	25.28/0.6881	27.93/0.8141	31.84/0.9154	32.66/0.9303	34.94/0.9559	36.94/0.9711	31.59/0.8780
SODAS-Net	24.69/0.7838	26.59/0.7528	30.62/0.8818	31.69/0.9052	33.67/0.9360	35.54/0.9568	30.46/0.8694
DPC-DUN	24.90/0.7856	26.79/0.7611	30.71/0.8828	31.76/0.9051	33.70/0.9364	35.62/0.9573	30.58/0.8713
AMAUN-CS	25.67/0.7921	27.96/0.8132	3 1. 92/0.9 161	33.62/0.9399	35.68/0.9611	37.71/0.9748	32.09/0.8995
AMAUN-CS^+^	**25.72/0.7945**	**28.10/0.8210**	**32.** **10/0.9** **213**	**34.** **20/0.9** **469**	**36.01/0.9630**	**37.85/0.9802**	**32.33/0.9044**

**Table 4:** Comparison of the parameters, running time, and FLOPs for reconstructing the Set11 dataset in the case of CS ratio = 10%. The best performance is labeled in bold.

Method	Running Time(s)	Params(M)	FLOPs(G)
ISTA-Net^+^	0.0188	1.112	405.12
AMP-Net	0.0564	1.529	434.84
OPINE-Net^+^	0.0156	1.095	384.34
TransCS	0.0286	1.916	564.86
AMAUN-CS	**0.0143**	**0.554**	**264** **.2** **0**
AMAUN-CS^+^	0.0172	1.103	396.70

**Table 5:** Ablation experiments of different modules on the Set11 dataset under a 50% sampling rate. The best PSNR (dB) is labeled in bold.

Cases	CAAGDM	LDGDM	DMTM	PSNR/SSIM
(a)	×	×	×	38.06/0.9706
(b)	√	×	×	40.12/0.9781
(c)	×	√	×	39.54/0.9742
(d)	√	√	×	41.00/0.9830
(e)	√	√	√	**41.15/0.9859**

## Data Availability

The BSD400 dataset used in the experiments can be found at https://www2.eecs.berkeley.edu/Research/Projects/CS/vision/bsds (accessed on 16 December 2024), the DIV2K dataset can be found at https://data.vision.ee.ethz.ch/cvl/DIV2K (accessed on 16 December 2024), the Set11 dataset can be found at https://github.com/KuldeepKulkarni/ReconNet (accessed on 16 December 2024), the Urban100 dataset can be found at https://github.com/jbhuang0604/SelfExSR (accessed on 16 December 2024), the BSD68 dataset can be found at https://github.com/cszn/DnCNN (accessed on 16 December 2024).
